# Reducing calibration time in motor imagery-based BCIs by data alignment and empirical mode decomposition

**DOI:** 10.1371/journal.pone.0263641

**Published:** 2022-02-08

**Authors:** Wei Xiong, Qingguo Wei

**Affiliations:** Dept. of Electronic Information Engineering, School of Information Engineering, Nanchang University, Nanchang, People’s Republic of China; Universiti Tunku Abdul Rahman, MALAYSIA

## Abstract

One of the major reasons that limit the practical applications of a brain-computer interface (BCI) is its long calibration time. In this paper, we propose a novel approach to reducing the calibration time of motor imagery (MI)-based BCIs without sacrificing classification accuracy. The approach aims to augment the training set size of a new subject by generating artificial electroencephalogram (EEG) data from a few training trials initially available. The artificial EEG data are obtained by first performing empirical mode decomposition (EMD) and then mixing resulting intrinsic mode functions (IMFs). The original training trials are aligned to common reference point with Euclidean alignment (EA) method prior to EMD and pooled together with artificial trials as the expended training set, which is input into a linear discriminant analysis (LDA) classifier or a logistic regression (LR) classifier. The performance of the proposed algorithm is evaluated on two motor imagery (MI) data sets and compared with that of the algorithm trained with only real EEG data (Baseline) and the algorithm trained with expanded EEG data by EMD without data alignment. The experimental results showed that the proposed algorithm can significantly reduce the amount of training data needed to achieve a given performance level and thus is expected to facilitate the real-world applications of MI-based BCIs.

## Introduction

A brain-computer interface (BCI) allows disabled people to communicate with the outside world and control external devices based on various neuroimaging technology such as electroencephalography (EEG), magnetoencephalography (MEG) or positron emission tomography (PET) [1–3]. Among them, EEG-based BCIs have received increasing attention due to its ease of use, low cost and high temporal resolution compared to MEG and PET [4, 5]. For example, a BCI based on EEG signals can be used to help paralyzed patients control wheelchair without the involvement of neural muscles. With the continuous development and progress of science and technology, the application of BCIs has even been expanded to healthy people, and is no longer limited to a specific field [6, 7].

Motor imagery (MI) is one of main paradigms for building a BCI whose input signals are generated by users via imagining their own limb movements or observing the movements of others [8, 9]. In neurophysiology, motor intention shows attenuation or enhancement of rhythmic activity in the specific frequency bands of μ rhythm (8–13 Hz) and β rhythm (14–30 Hz) over the sensorimotor cortex. The two physiological phenomena are termed event-related desynchronization (ERD) and event-related synchronization (ERS) respectively. They can be interpreted as the mental rehearsal of a motor action without indeed execution of movement. Since a MI-based BCI does not need any external stimulation, MI is a more natural way to construct a BCI than other BCI paradigms.

Since EEG signals have low signal-to-noise ratio (SNR), complex signal processing and machine learning algorithms are needed to decode the intentions which the users want to express [10]. Before decoding, the EEG signals are pre-processed through band-pass filters for removing artifacts and noise unrelated to the classification of mental tasks. Typically, multiple electrode channels are adopted for recording EEG signals in order to extract enough information from brain activities arisen by mental tasks. Therefore, an effective spatial filtering technique is needed to integrate EEG signals so that their SNR can be further improved and discriminative information can be extracted for subsequent classification task.

Because large variability exists across subjects and across sessions of the same subject, the classification model needs to be rebuilt from scratch of each use. Thereby, one of the major problems in the development of a BCI is its long calibration time. This problem severely limits the real-world application of BCI systems. To address the problem, researchers have presented four main types of approaches that are based on regularization [11, 12], user-to-user transfer [13–16], semi-supervised learning [17–19] and a prior physiological information [20, 21]. Recently, a novel approach was proposed for reducing calibration time that is to generate artificial EEG data from a few actual training data available and use them to augment the training set.

Lotte proposed to generate artificial data by signal segmentation and recombination [22, 23]. This simple approach enables us to yield numerous artificial trials, different from the original ones, but still relevant and likely to be similar to future trials, as they are made from parts of real trials and have the same temporal structure. But this method only preserves the time characteristic of the signal and cannot control the frequency characteristic. Wang et al. added Gaussian white noise to the training data in the time domain to obtain new samples for an emotion-recognition task [24]. The authors opted for Gaussian noise due to concerns that adding some local noise (i.e., noise that affects EEG data locally) such as Poisson or salt-and-pepper may change the intrinsic features of EEG signals. Zhang et al. proposed a conditional deep convolutional generative adversarial network (cDCGAN) to generate numerous artificial data [25]. Using data generated from the cDCGAN, classification accuracy increased from 83% to 86%. Dinares-Ferran et al. proposed a new method to create artificial EEG frames using empirical mode decomposition (EMD) and mixing their intrinsic mode functions (IMFs) for a MI-based BCI [26]. They tested the performance of the algorithm on an internal and an external MI data set. In the case that the EEG trials containing artifacts and outliers were removed by median absolute deviation (MAD), good classification results were achieved on two of the seven subjects in internal data set and two of four subjects in external data set.

Motivated by above-mentioned studies, we proposed a novel algorithm for reducing calibration time that generates artificial EEG data from a small amount of training trials by combining the two algorithms of data alignment (DA) and EMD. Different from the algorithm developed by Dinares-Ferran et al., EMD is applied to aligned data instead of raw data and to whole EEG trials instead of the EEG trials with artifacts and outliers removed. Common spatial pattern (CSP) is adopted for feature extraction and either linear discriminant analysis (LDA) or logistic regression (LR) for classification. The performance of the proposed algorithm was evaluated on two publicly available BCI data sets and compared with those of baseline algorithm without using artificial trials and the algorithms without applying data alignment.

## Methods

In this section, we introduce the classification pipeline for reducing calibration time in MI-based BCIs, including the concepts such as empirical mode decomposition (EMD), artificial EEG frames generation (AFG), data alignment (DA), common spatial pattern (CSP), and two classifiers, i.e. linear discriminant analysis (LDA) and linear regression (LR). It is noted that frame and trial are two replaceable words in this paper because an EEG frame corresponds to an EEG trial.

### Empirical mode decomposition

Because EEG signals are non-linear and non-stationary, commonly used analytical tools like FFT and wavelets would not be adequate to process EEG data. The EMD proposed by Huang et al. [27] provides a method for addressing the problem, which allows users to conduct a data-driven analysis to better fit with non-stationary signals that have changes in the frequency structure within a short period of time. EMD has been widely used in the field of brain-computer interfaces [28–31]. The method decomposes an original signal into a finite number of functions called intrinsic mode functions (IMFs), each of which represents a non-linear oscillation of the signal. These IMFs fulfill two conditions: 1) In the whole signal, the number of maxima is the same as the number of zero-crossing, or differs by at most one; 2) For any sample, the mean value between the envelope of the local maxima and the envelope of the local minima is zero. The processing procedures of EMD for obtaining the IMFs from a signal *x*(*t*) are as follows:

1. Set *s*(*t*) = *r*_0_(*t*), initialize *r*_0_(*t*) = *x*(*t*), *i* = 1.
2. Detect the local maxima and the local minima of *s*(*t*).
3. Interpolate all local maxima and all local minima to get the upper envelope and the lower envelope, respectively.
4. Calculate the local mean *m*(t) by averaging the upper and the lower envelopes.
5. Get a new signal as a candidate IMF by *h*(*t*) = *s*(*t*)−*m*(*t*).
6. Decide whether *h*(*t*) satisfies the IMF’s conditions. If the conditions are not satisfied, begin a new loop from step 2 and set *s*(*t*) = *h*(*t*).
7. If the conditions are satisfied, *h*(*t*) can be defined as an IMF: *IMF*_*i*_(*t*) = *h*(*t*).
8. Obtain *r*_*i*_(*t*) by subtracting the IMF from *r*_*i*−1_(*t*): *r*_*i*_(*t*) = *r*_*i*−1_(*t*)−*IMF*_*i*_(*t*).
9. If *r*_*i*_(*t*) satisfies the stopping criterion, then the decomposition ends.
10. Otherwise, set *s*(*t*) = *r*_*i*_(*t*) and begin a new loop from step 2 to get *IMF*_*i*+1_(*t*).

In practice, the above procedures have to be refined by a sifting process that amounts to iterating steps 2) to 10) upon the detail signal *h*(*t*), until this latter can be considered as zero-mean according to some stopping criterion, i.e. *r*_*i*_(*t*) is a monotonic function or does not have enough extrema to calculate the upper and lower envelopes [27, 32–34]. The resulting IMFs are ranked according to frequency from high to low. Once all the IMFs have been calculated, the signal can be recovered by Eq (1) using all its IMFs and the final residue *r*_*n*_(*t*), where *n* is the number of extracted IMFs.


$$x(t)=\sum_{k=1}^{n}imf_{k}(t)+r_{n}(t)$$ (1)

### Generation of artificial EEG frames

Using the EMD approach, the artificial EEG frames can be created by combining some IMFs from different real EEG frames. First of all, the generated artificial EEG trials exhibit similar characteristics in time and frequency to the real EEG trials used for yielding these IMFs, due to the fact that each IMF represents a specific non-linear oscillation. In this paper, the dimension of the original training set is *N*_*S*_×*N*_*C*_×*N*_*T*_, where *N*_*S*_, *N*_*C*_ and *N*_*T*_ are the number of sampling points in a trial, the number of EEG channels and the total number of EEG trials respectively. Because the number of channels affects the performance and computational speed of the proposed classification algorithms, we manually selected the 15 electrode channels located in the central cortex of the brain, including C3, C4, Cz and the nearest set of channels based on prior knowledge of neurophysiology [33], in order to reduce time consumption as well as the effect of noise from irrelevant channels. Therefore, we manually select 15 electrode channels located in the central cortex of the brain, including C3, C4, Cz and the nearest set of electrode channels. Accordingly, the artificial EEG trials have the same number of channels as real EEG trials, i.e. 15 channels. An artificial EEG trial is generated according to the following steps:

1. Select the same number of real EEG trials from each class of a subject according to the order in a data set to generate their IMFs;
2. Specify the number of artificial frames per class to be created as the multiple of the chosen real EEG frames per class;
3. Randomly select a subset from the chosen real EEG trials in step 1) and determine all IMFs according to the order of randomly chosen trials in the subset. Each IMF contains 15 signals corresponding to 15 channels;
4. Generate an artificial EEG trial, each channel signal of which is yielded by adding all five IMFs of the same channel.

Repeat step 3)-4) to generate a new artificial EEG trial until the specified number of artificial trials in step 2) is created.

In this study, the first five IMFs, including 15 channels of signals each, were used for artificial frame synthesis. The reason is that the decomposed IMFs were arranged from high to low according to the amount of information contained, in line with the characteristics of EMD algorithm. In addition, previous studies [26] indicate that the information related to mental tasks of MI is mainly scattered in the first five IMFs, so we used them as the IMF set of each channel signal.

We used this procedure to create artificial frames for augmenting the training set of each subject. Each training set contains artificial frames with different multiples of the real frames, the multiples varying from 0 to 10. This process created 11 collections of EEG training frames, the first of which corresponds to the original training set and thus has no artificial frames. Based on each of the 11 frame collections, a classifier is constructed and its classification accuracy is determined accordingly.

### Data alignment

Due to the randomness and non-stationarity of EEG signals, there is great difference in data distribution between sessions and even within a session. This difference will result in a much greater difference in data distribution between real EEG trials and the artificial EEG trials created from them. Data alignment (DA) aligns EEG trials or their covariance matrices to a common reference and thus is a valid method for reducing their difference in distribution [35–37]. Thereby, it is very beneficial to perform data alignment before the creation of artificial data so that the real EEG trials are more consistent in distribution. Several DA approaches exist in literature such as RA [35], EA [36] and PS [37], depending upon how the reference matrix is defined. Euclidean alignment (EA) [36] is adopted in the study because it can be used as either a supervised or a unsupervised method.

In EA, the reference matrix is obtained by averaging all EEG trials from two classes without the need of label information. The basic principle of EA is to align single-trial EEG signals one by one with the reference matrix and move them toward the reference matrix, making the distribution of EEG data from different sessions or subjects more similar. Assume a subject has total *I* EEG trials including two classes, $X_{i}\in R^{N_{C}\times N_{S}},i=1,2,\cdots ,I$, the reference matrix is calculated as

$$\bar{R}=\frac{1}{I}\sum_{i=1}^{I}X_{i}X_{i}{}^{T}$$ (2)

Then each of the *I* EEG trials is aligned as

$${\tilde{X}}_{i}={\bar{R}}^{-1/2}X_{i}$$ (3)

The mean covariance matrix of the *n* aligned trials is equal to an identity matrix, so these covariance matrices are distributed near the identity matrix while their relative distances remain unchanged.

### Feature extraction and classification

In a machine learning algorithm, classification tasks are usually divided into two main parts. The first part is the extraction of related features, which aims to extract the class invariant features in the tasks, whereas the second is the classification of feature signals, which uses the specific algorithm to classify the extracted features. CSP is a powerful algorithm for spatial filtering and subsequent feature extraction in MI-based BCIs [38, 39]. LDA is a typical feature discrimination method. It is adopted in the study because of its robustness to noise and having no adjustable parameters [40, 41]. LR is a classifier for building linear logistic regression models [42]. It is adopted in this study because the classifier directly models the probability of classification without the need to meet the hypothetical data distribution.

#### Feature extraction

The CSP aims to find a spatial filtering matrix $W\in R^{N_{C}\times N_{C}}$ that maximizes the variance of one class while minimizes the variance of the other class [38, 39]. By jointly diagonalizing the two classes of signals, CSP enhances the difference in variance (or band power) between two conditions. The spatial filtering matrix (also called projection matrix) *W* is obtained by maximizing or minimizing the following cost function:

$$J(W)=\frac{W^{T}{\bar{C}}_{1}W}{W^{T}{\bar{C}}_{2}W}$$ (4)

where ${\bar{C}}_{y},y=1,2$ is the average spatial covariance matrix of class *y* from band pass filtered EEG signals, and *T* represents a transpose operation. In general, the average spatial covariance matrix is obtained by first calculating the spatial covariance matrix *C*_*y*_^*j*^ of *j*th training trial *T*_*y*_^*j*^ of class *y*, and then taking their average:

$${\bar{C}}_{y}=\frac{1}{N_{y}}\sum_{j}^{N_{y}}C_{y}{}^{j}=\frac{1}{N_{y}}\sum_{j}^{N_{y}}T_{y}{}^{j}{\left(T_{y}{}^{j}\right)}^{T}$$ (5)

*N*_*y*_ is the number of training trials of class *y*; $T_{y}{}^{j}\in R^{N_{C}*N_{S}}$ is the *j*th trial of class *y*, where *N*_*C*_ is the number of EEG channels in the trial, and *N*_*S*_ is the number of sampling points. The optimal projection matrix $\tilde{W}$ is composed of the eigenvectors of ${\bar{C}}_{2}{}^{-1}{\bar{C}}_{1}$, which corresponds to its *m* largest and *m* smallest eigenvalues (*m* = 3 in the study). The *p*th column of $\tilde{W}$, ${\tilde{w}}_{p}\in R^{N_{C}\times 1}$, is called a spatial filter, whereas ${\tilde{w}}_{p}^{-1}$ is called a spatial pattern. Once the projection matrix is obtained, the feature vector f∈*R*^2*m*^ used for classification is extracted by

$$f=\log(\tilde{W}C_{ct}{\tilde{W}}^{T})$$ (6)

where *C*_*ct*_ is a single-trial covariance matrix.

#### Classification

1) The LDA classifier separates feature vectors using a linear hyper-plane [40, 41]. With LDA, the classification score (i.e., the output of the classifier before taking the operation of sign) of an input feature vector f is equal to

$$s=a^{T}f+b$$ (7)

where the normal vector a and intercept *b* are respectively calculated as follows:

$$a^{T}=(C^{-1}){\left(m1-m2\right)}^{T},\;b=-\frac{1}{2}(m1+m2)a^{T}$$ (8)

where m1 and m2 are the mean feature vectors from class 1 and class 2 respectively, and *C* is the mean covariance matrix of two-class feature signals. If the score *s* = a^*T*^ f+*b* is positive, the feature vector is assigned to class 1, and vice versa, to class 2.

2) The LR is a classifier for building linear logistic regression models [38]. Given feature data f and weights (w, *b*), the linear regression model can be expressed as:

$$P(y=\pm 1|f,w)=1/(1+e^{-y(w^{T}f+b)})$$ (9)

where *y* is the class label. If there are *I* training instances f_*i*_,*i* = 1,2,⋯,*I*, and the labels are *y*_*i*_∈{1,−1}, the weights (w, *b*) can be estimated by minimizing the negative log-likelihood:

$$\underset{w,b}{\min}\sum_{i=1}^{I}{\log(1+e}^{-y_{i}(w^{T}f_{i}+b)})$$ (10)

Given a feature vector f from a testing trial, it is assigned to class 1 if the score *z* = w^*T*^ f+*b* is positive and to class 2 otherwise.

### The proposed classification pipeline

**Fig 1:**
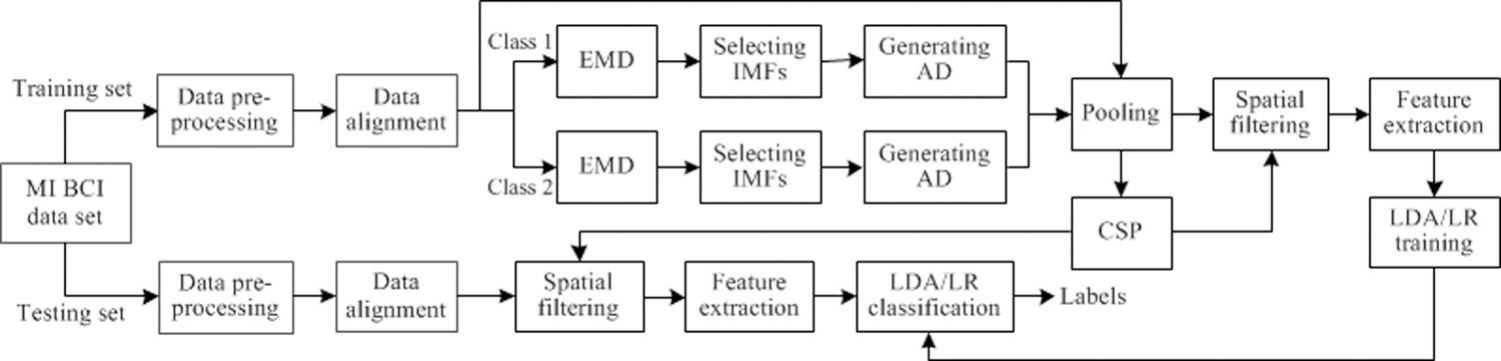
Algorithmic flowchart for classifying MI-based EEG signals using the proposed data augmentation algorithm based on CSP and LDA or LR. AD denotes artificial data.

The MI-based BCI data set from a subject is divided into a training set and a testing set, which are preprocessed by data segmenting and bandpass filtering, and then aligned by EA approach. Then the aligned training set is further divided into two classes of EEG trials, which are separately subject to EMD, selection of IMFs and generation of artificial data. Next the artificial EEG trials from two classes and the aligned original training trials pooled together as the expended training set, which is put into CSP algorithm for estimating spatial filters. Both the expended EEG trials and the aligned testing trials are filtered by the estimated spatial filters and then feature extraction is conducted based on the spatially filtered data. The feature signals obtained from the expended training set are used for training a LDA or a LR classifier, which is used for classifying feature signals from testing set. The classification pipeline of the proposed algorithm is illustrated in Fig 1.

## Experimental data

In this study, two MI-based EEG data sets were used for evaluating the performance of the proposed algorithm. The first one is the data set 2a of BCI Competition IV [43, 44], and the second is the data set 4a of BCI Competition III [45, 46]. The two data sets were adopted because they are most widely used to compare different classification algorithms.

### Data set 1

**Fig 2:**
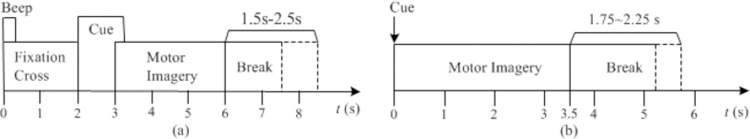
The timing scheme of a single trial in (a) the EEG dataset 1 and (b) the EEG dataset 2.

The data set contains EEG data from 9 healthy subjects (named as A1, A2,⋯,A9). The cue-based BCI paradigm consisted of four different mental tasks, namely MI of left hand, right hand, both feet and tongue, which are indicated by arrows in the left, right, down, or up directions, respectively. During the experiment, the subject was sitting comfortably in front of the computer screen. At the beginning of a trial, a fixation cross and a short acoustic warning tone prompted the subject to be prepared. After two seconds, an arrow cue from one of four directions appeared and stayed on the screen for 1.25 s which prompted the subjects to perform the desired MI task. The mental imagination lasted until 6 s. No feedback was provided. A short break followed that lasted 1.5 s-2.25 s. The timing scheme of each trial is shown in Fig 2(A). The EEG data were recorded using 22 Ag/AgCl electrodes and sampled at 250 Hz. Each subject performed two sessions (T and E) on different days. Each session includes six runs separated by short breaks. One run is composed of 48 trials, 12 trials per task, yielding a total of 288 trials per session. The two classes of EEG data from left and right hand in each session were used in the study.

### Data set 2

The data set contains EEG data from 5 healthy subjects (named as *S*1, *S*2,⋯,*S*5). The cue-based BCI paradigm consisted of three different mental tasks, namely MI of left hand, right hand, and right foot. During the experiment, the subject was sitting comfortably in front of the computer screen. Visual cues indicated for 3.5 s, during which the subject should perform one of the three MI tasks. The presentation of target cues is intermitted by periods of random length, 1.75 to 2.25 s, in which the subject could relax. This data set contains only data from the 4 initial sessions without feedback. The timing scheme of each trial is shown in Fig 2(B). The EEG data were recorded using 118 Ag/AgCl electrodes in line with international 10/20 system and sampled at 1000Hz. Only EEG data from MI of ’left hand’ and ’ right foot’ were provided for the competition. Each subject has a total of 280 trials, 140 trials per class.

### Data preprocessing

For the classification of mental tasks, each trial of the subjects in the two data sets was band-pass filtered by a 5th order Butterworth filter in the frequency band of 8–30 Hz. The data segments of 2 s were intercepted from 2.5s to 4.5s and from 0.5s to 2.5s for the data set 1 and 2 respectively.

## Results

This section systematically analyzes the proposed algorithm from four aspects, i.e. EMD basic decomposition process, data visualization, number of training trials, and classification performance. It is noted that for the data set 1, the first session (T) was used as training set and the second one (E) as the testing set, whereas for the data set 2, the first half of the single session is used as training set and the second half of the session as the testing set. The training trials per class used for generating artificial trials were selected sequentially from the training set in order to imitate the real online experimental situation.

### EMD process

**Fig 3:**
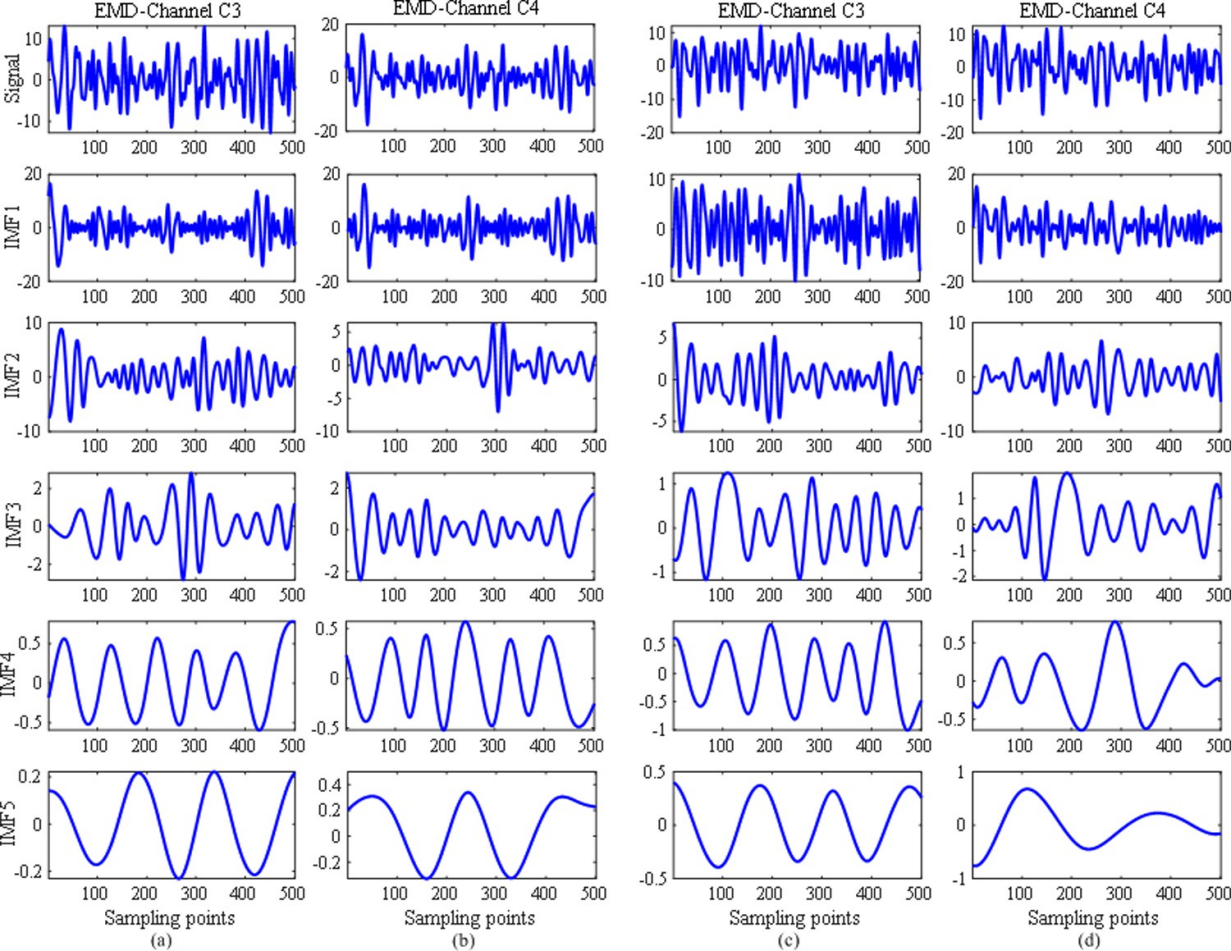
(a) and (b) The two EEG signals and their respective five IMFs induced by MI of left hand, acquired from channels C3 and C4, trial 1 of subject A01T in data set 1; (c) and (d) The two EEG signals and their respective five IMFs induced by MI of right hand, acquired from channels C3 and C4, trial 1 of subject A01T in data set 1.

In order to explain the principle of the EMD method, we used two EEG signals per class from subject A01T in data set 1 to obtain IMFs. The two signals were acquired from channels C3 and C4. The two signals and their respective five IMFs induced by MI of left hand are showed in Fig 3(A) and 3(B) respectively, whereas those induced by MI of right hand are shown in Fig 3(C) and 3(D) respectively. As shown in the two columns in Fig 3(A) or 3(B), the first IMF was generated by EMD of the original signal, and was used to gradually obtain the other IMFs. Specifically, the second IMF was obtained by subtracting the first IMF from the original signal and the remaining IMFs were achieved by subtracting the previous generated IMFs from the original signal. It is easily observed that the first few IMFs fluctuate more remarkably than others. That means that the main information of the original signal is generally distributed in the first few IMFs. Accordingly, in the study we only used the first five IMFs to generate artificial EEG data for augmenting the original training data set.

### Data visualization

**Fig 4:**
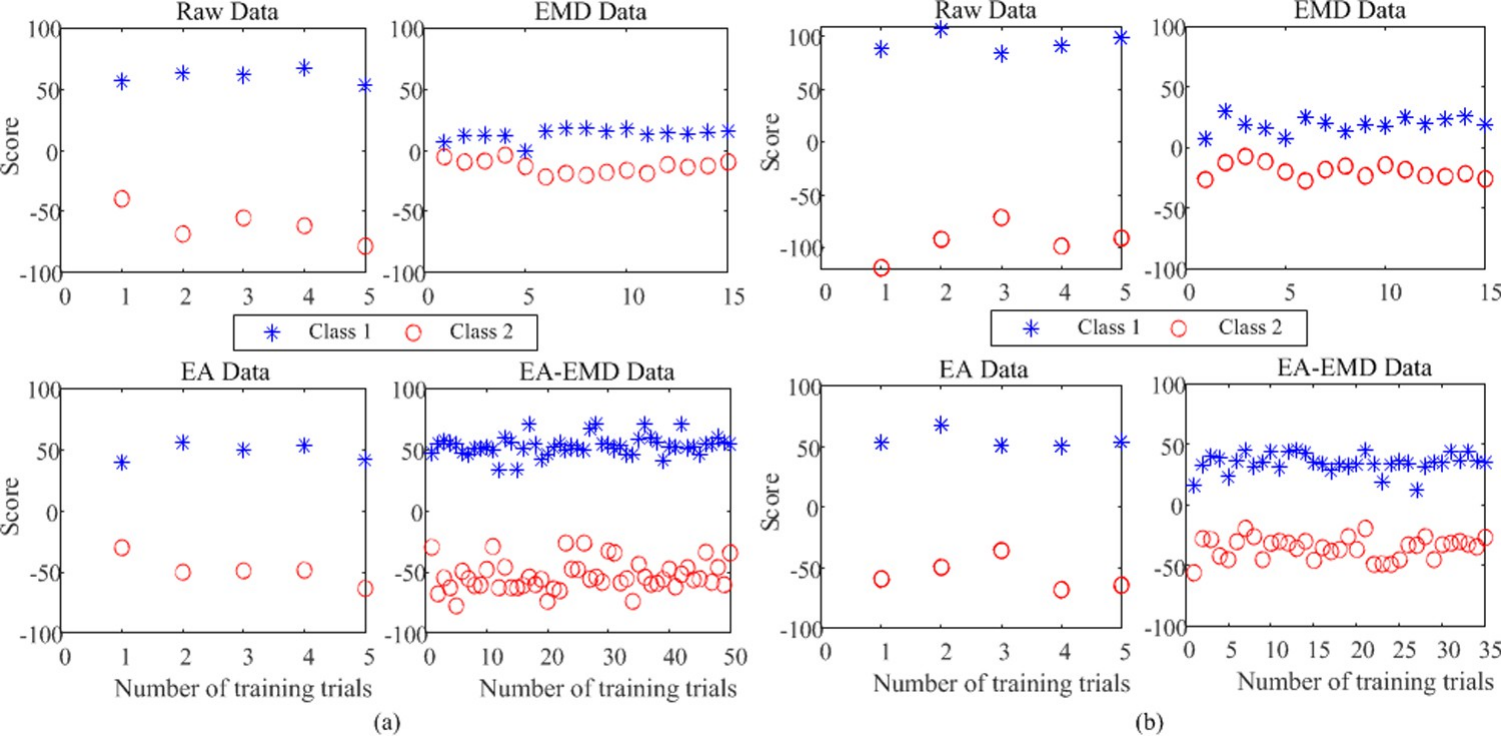
Taking five raw training trials per class as an example for building the classification model, the classification scores of raw data, EA data, EMD data, and EA-EMD data derived from (a) subject A3T in data set 1 and (b) subject S5 in data set 2. EMD data and EA-EMD data are the expended data including artificial data generated by EMD in the case of the best multiple of artificial frames, which are 2 and 9 respectively for Fig 4(A) respectively and 2 and 6 respectively for Fig 4(B).

To display the difference in feature distribution among raw data, EA data, EMD data, and EA-EMD data, we take five raw training trials per class as an example for building the classification model. Each feature vector extracted by Eq (6) is reduced to a scalar value by Eq (7) and thus can be represented by a scalar value. Fig 4(A) and 4(B) illustrate the feature distributions of the four types of training data for subject A3T in data set 1 and subject S5 in data set 2 respectively. EMD data and EA-EMD data are the expended data including artificial data generated by EMD in the case of the best multiple of artificial frames on the basis of raw and EA data respectively.

From the figure, it is observed that 1) Compared to raw data, the difference in feature distribution of EA data is decreased to some extent; 2) EMD data enrich the feature space by adding artificial data and thus increase the variability of training data. This is expected to help the subsequent classifier cope with variant features from testing signals. However, the averaged distance between two classes of feature signals became much shorter for the two data sets and even there were a few trials difficult to separate. 3) Compared to EMD data, EA-EMD data further increase the variability of training data by adding more artificial frames and the separability between the two classes of feature signals is excellent. These observations indicate that applying EMD to raw data may not a valid method for creating artificial data, but applying EMD to EA data indeed improve the classification performance of MI-based BCIs.

### Number of training trials

**Fig 5:**
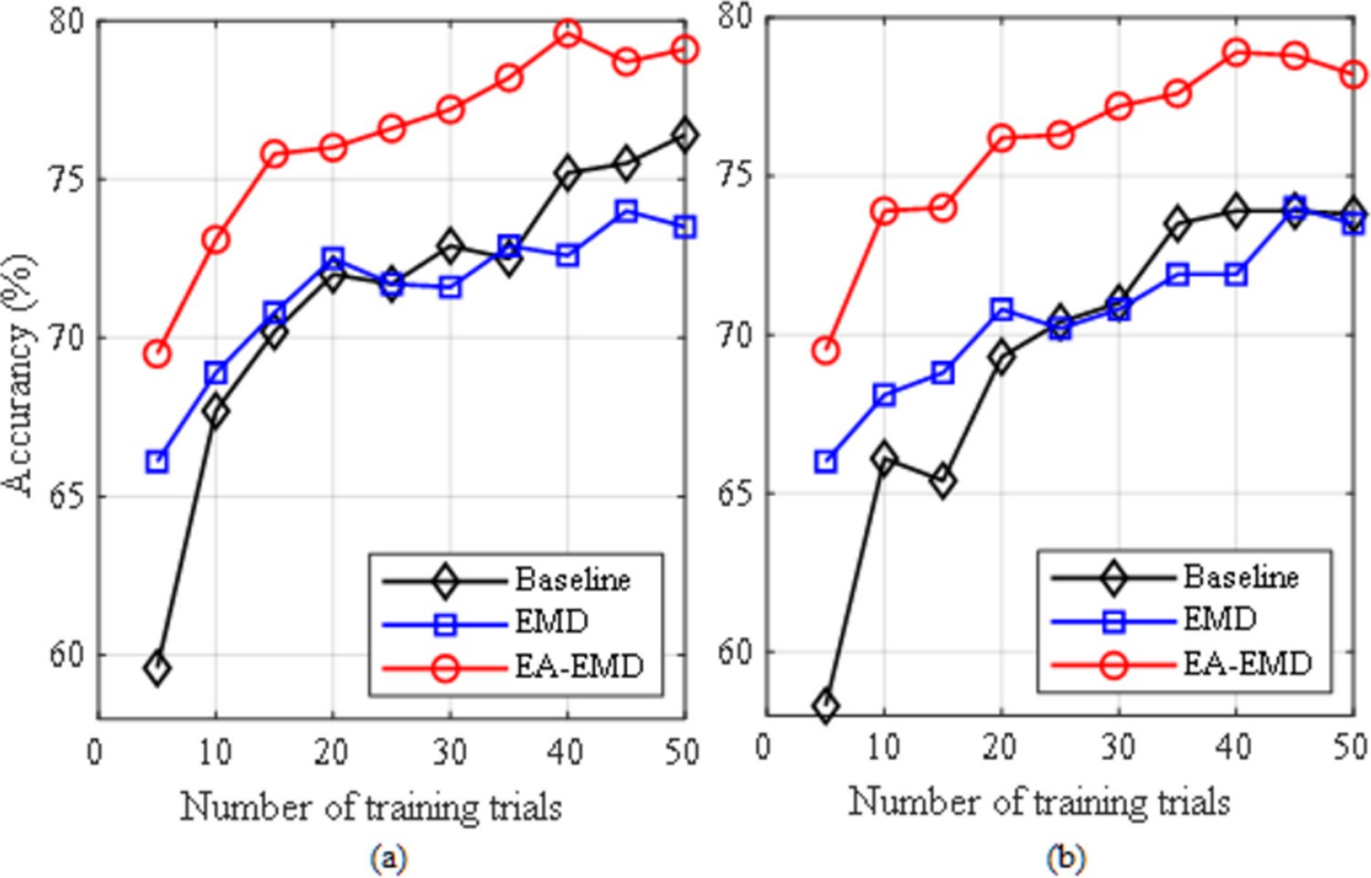
Averaged accuracies across all subjects in dataset 1 yielded by the three classification pipelines using (a) LDA and (b) LR as classifier.

**Fig 6:**
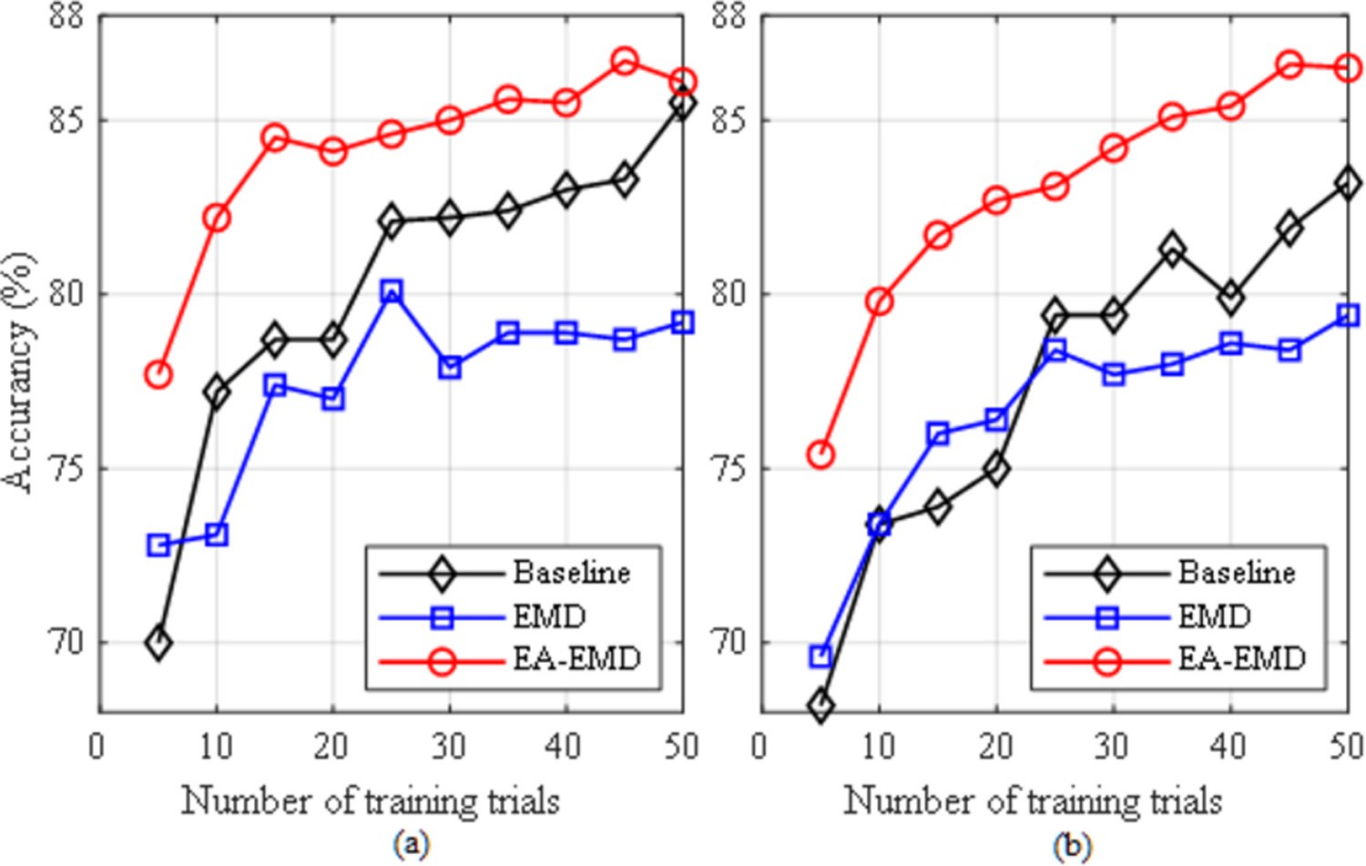
Averaged accuracies across all subjects in dataset 2 yielded by the three classification pipelines using (a) LDA and (b) LR as classifier.

In the study, CSP+LDA and CSP+LR were applied to the following three types of data for performance comparison: 1) the real training data (Baseline); 2) the augmented training data by EMD only (EMD); 3) the augmented training data by EA-EMD (EA-EMD). The evaluation criterion for these algorithms is classification accuracy, which is defined as the ratio of the number of correctly classified testing trials to the total number of testing trials. In this subsection, we studied the performance of these algorithms based on different numbers of training trials, starting from the first 5 training trials per class and then increasing at the interval of 5 trials until 50 trials. The experimental results yielded by classifiers LDA and LR for data set 1 are shown in Fig 5(A) and 5(B) respectively, whereas those for data set 2 are shown in Fig 6(A) and 6(B) respectively. The results from the latter two methods were based on the best multiples of artificial frames.

From the two figures, it can be observed that regardless of data sets and classifiers, EA-EMD outperformed the other two algorithms for all numbers of training trials per class. For data set 1, EMD yielded better accuracies than Baseline when the number of training trials was less than 25 using either classifier, whereas for data set 2, EMD yielded better accuracy only at 5 training trials using LDA and better accuracies when the number of training trials was less than 25 using LA. As a direct comparison of calibration time, for data set 1, the accuracies of EA-EMD yielded by ten training trials per class was equal to those of Baseline yielded by 30 and 40 training trials with LDA and LR respectively, reducing the calibration time by 2/3 and 3/4; For data set 2, the accuracies of EA-EMD yielded by ten training trials per class was equal to those of Baseline yielded by 30 and 30 training trials with LDA and LR respectively, reducing the calibration time by 2/3 and 2/3 respectively.

Paired *t*-tests were conducted on classification accuracies at each number of training trials in Figs 5 and 6 respectively to compare any two of the three algorithms. In all the *t*-tests, the significance level was set as *α* = 0.05. The results of the statistical tests are listed in Tables 1 and 2 respectively. From the two tables, it is observed that using LDA, EA-EMD is significantly better than Baseline when the number of training trials is less than 30, whereas using LR, EA-EMD is significantly better than Baseline at all numbers of training trials. There are no significant differences between EMD and Baseline at all numbers of training trials regardless of classifiers.

**Table 1:** *p* values of paired t-tests at 95% confidence level among the three algorithms Baseline (M1), EMD (M2) and EA-EMD (M3) based on two classifiers LDA and LR for data set 1.

Number of training trials	LDA			LR		
	M3-M1	M3-M2	M2-M1	M3-M1	M3-M2	M2-M1
5	**0.020**	**0.005**	0.105	**0.020**	0.208	0.149
10	**0.018**	**0.017**	0.615	**0.029**	**0.004**	0.579
15	**0.021**	0.128	0.880	**0.021**	0.051	0.336
20	**0.005**	0.117	0.834	**0.001**	**0.025**	0.565
25	**0.010**	**0.028**	0.996	**0.014**	**0.039**	0.928
30	0.087	**0.010**	0.610	**0.027**	**0.013**	0.947
35	**0.007**	**0.004**	0.823	**0.012**	**0.010**	0.286
40	**0.011**	**0.015**	0.373	**0.005**	**0.007**	0.388
45	0.081	**0.008**	0.372	**0.008**	**0.007**	0.970
50	0.086	**0.030**	0.197	**0.015**	**0.016**	0.929

**Table 2:** *p* values of paired t-tests at 95% confidence level among the three algorithms Baseline (M1), EMD (M2) and EA-EMD (M3) based on two classifiers LDA and LR for data set 2.

Number of training trials	LDA			LR		
	M3-M1	M3-M2	M2-M1	M3-M1	M3-M2	M2-M1
5	**0.010**	0.174	0.161	**0.014**	**0.037**	0.361
10	**0.017**	**0.067**	0.262	**0.105**	0.067	0.971
15	**0.034**	0.096	0.518	**0.001**	0.065	0.299
20	**0.031**	0.082	0.390	**0.005**	**0.054**	0.498
25	**0.007**	0.077	0.307	**0.017**	0.097	0.634
30	0.075	0.068	0.245	**0.013**	0.060	0.447
35	**0.025**	**0.034**	0.169	**0.016**	**0.035**	0.182
40	0.159	**0.027**	0.006	**0.008**	**0.015**	0.516
45	**0.012**	**0.049**	0.174	**0.008**	**0.037**	0.364
50	0.089	**0.051**	0.056	**0.009**	0.087	0.318

### Classification accuracy

**Fig 7:**
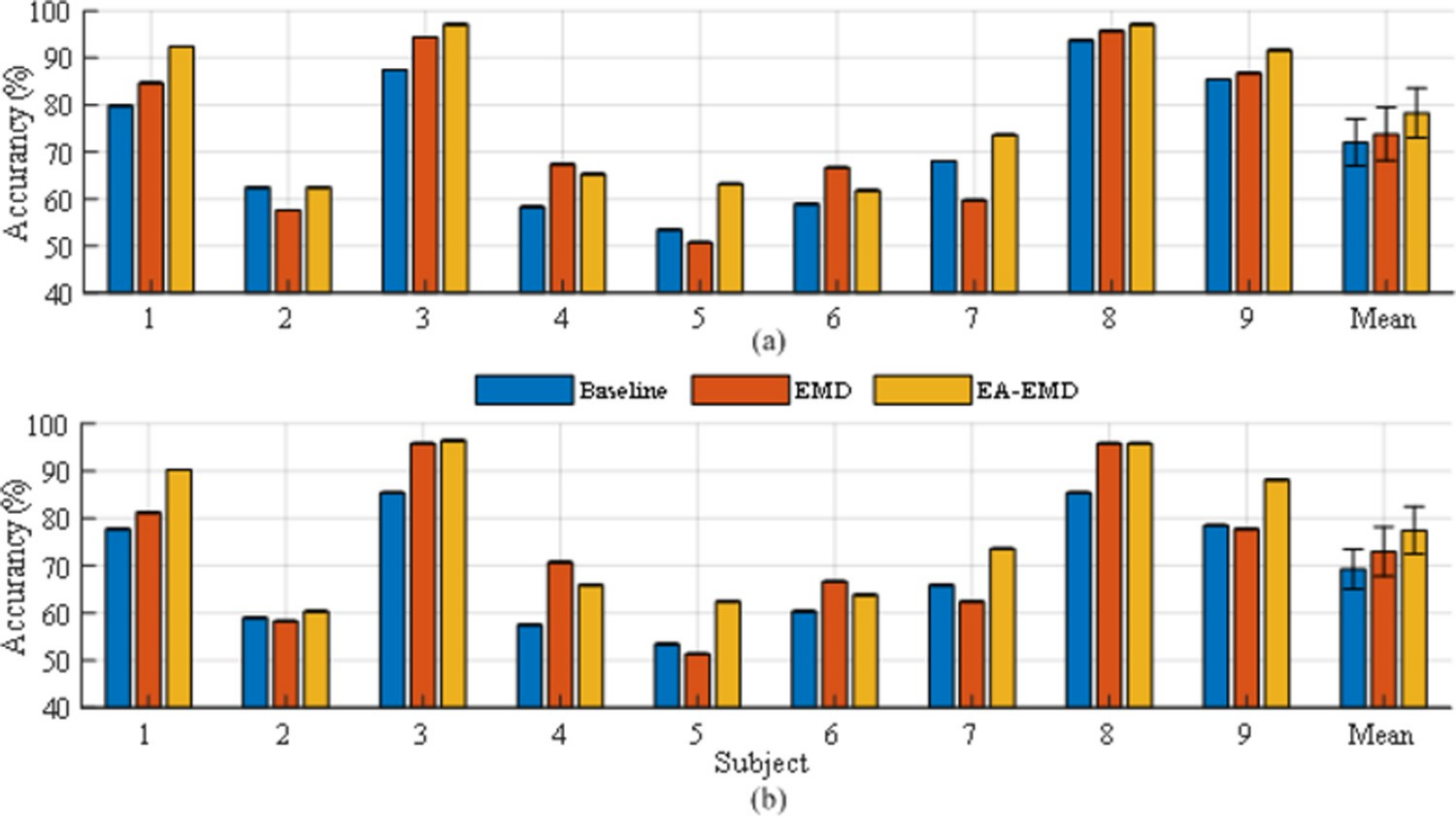
Classification accuracies of the nine subjects in data set 1 achieved by 20 training trails per class using (a) LDA and (b) LR as the classifier. The error bars represent standard errors.

**Fig 8:**
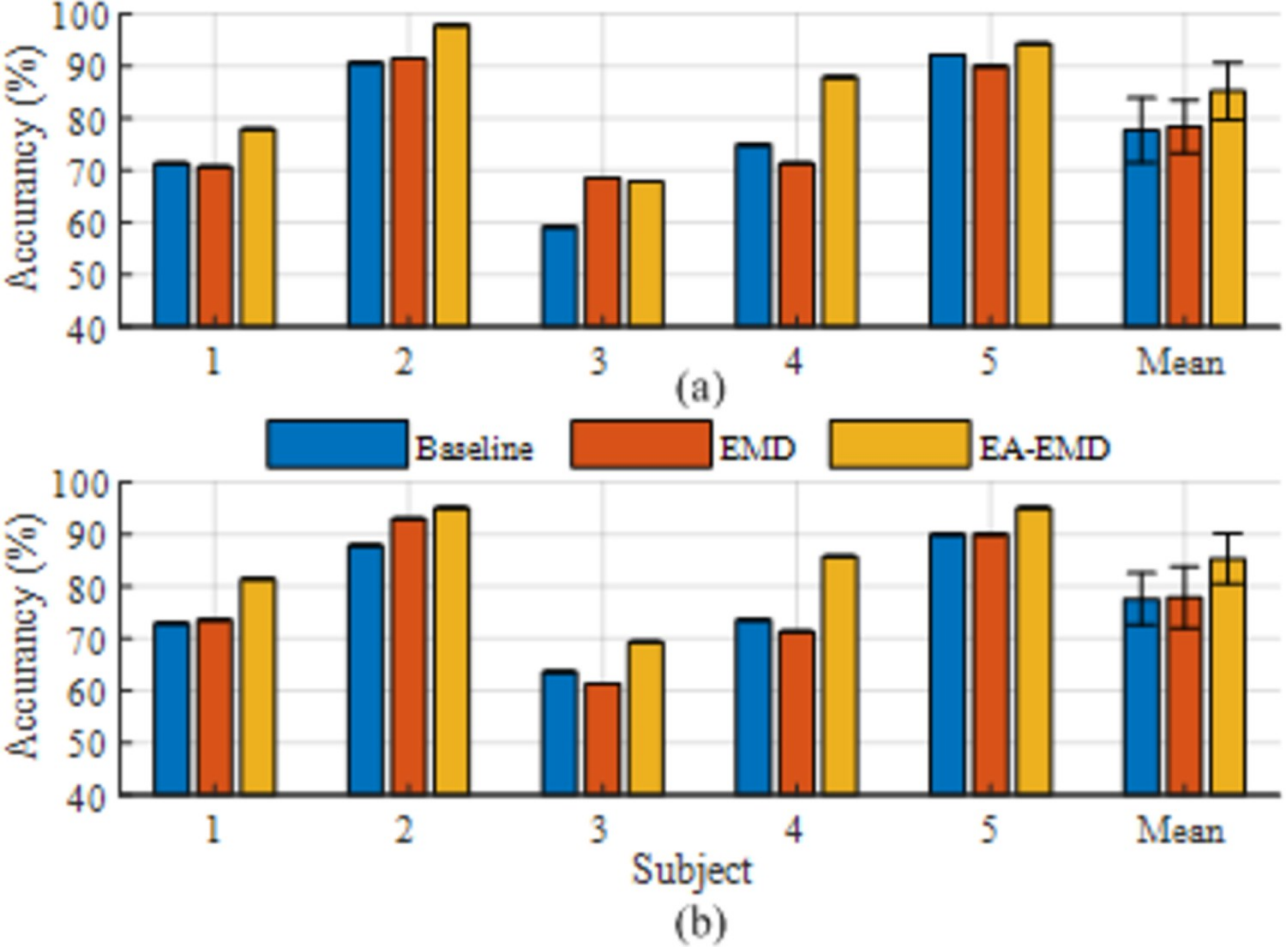
Classification accuracies of the five subjects in data set 2 achieved by 20 training trails per class using (a) LDA and (b) LR as the classifier. The error bars represent standard errors.

Taking 20 training trials per class as an example, the classification accuracies achieved by the three algorithms for the nine subjects in data set 1 are illustrated in Fig 7(A) and 7(B) with LDA and LR as classifiers respectively, whereas those for the five subjects in data set 2 are illustrated in Fig 8(A) and 8(B) with LDA and LR as classifiers respectively.

It is observed from Fig 7 that regardless of the classifier, EA-EMD achieved the highest accuracies on 6 of 9 subjects, whereas EMD achieved the highest accuracies on only two subjects. Using LDA as the classifier, the averaged accuracy across all subjects yielded by EA-EMD is 3.5%, higher than that yielded by EMD and 4% higher than that yielded by Baseline; Using LA as the classifier, the averaged accuracy across all subjects yielded by EA-EMD is 5.4% higher than that yielded by EMD and 6.9% higher than that yielded by Baseline. It is observed from Fig 8 that using either classifier, EA-EMD obtained the highest accuracies on all 5 subjects, whereas EMD did not obtain the highest accuracy. On average, using LDA, the accuracy yielded by EA-EMD is 7.1% and 5.4% higher than that yielded by EMD and Baseline respectively, whereas using LR, the accuracy yielded by EA-EMD is 6.5% and 7.9% higher than that yielded by EMD and Baseline respectively.

**Fig 9:**
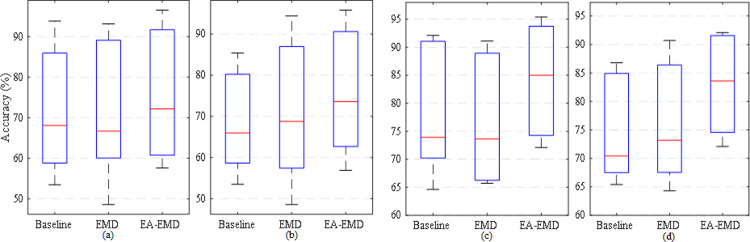
The maximal, the minimal and the median value of the averaged classification accuracies across all subjects in each or the two data sets yielded by the three algorithms Baseline, EMD and EA-EMD for (a) data set 1 and classifier LDA, (b) data set 1 and classifier LA, (c) data set 2 and classifier LDA, and (d) data set 2 and classifier LA.

For the three algorithms used for comparison in the paper, the box plots of accuracies yielded by the two classifiers and the two data sets are illustrated in Fig 9. From the figure, it is observed that irrespective of classifiers and data sets, the median of the averaged classification accuracies across all subjects in each of two data sets yielded by EA-EMD is consistently larger than those yielded by both Baseline and EMD.

### Multiples of artificial frames

Tables 3 and 4 report the best multiples of artificial frames generated by EMD and EA-EMD at each number of training trials for data set 1 and 2 respectively. In terms of data set 1, it is observed from Table 3 that irrespective of classifiers, the best multiples of artificial frames yielded by EMD range from 1 to 5, whereas those yielded by EA-EMD range between 6 and 10. With respect to data set 2, it can be seen from Table 2 that the situation is the same as data set 1 for EA-EMD, but the best multiples of artificial frames yielded by EMD range only from 1 to 3. At the same numbers of training trials, we compared the accuracies yielded at different multiples of artificial frames in the above-mentioned range and found that the difference in accuracy is less than 2% in most cases, meaning that the parameter is not very sensitive for classification of MI data. These results demonstrate that EA-EMD is able to expand more artificial frames than EMD and achieve the better classification accuracy than EMD. Thereby, EA-EMD is a feasible method for dealing with the problem of small sample data set and reducing the calibration time.

**Table 3:** Best multiples of artificial frames yielded by EMD and EA-EMD at each number of training trials for dataset 1.

Number of training trials	EMD		EA-EMD	
	LDA	LR	LDA	LR
5	2	3	9	9
10	4	4	10	10
15	3	3	6	10
20	2	2	9	9
25	2	4	8	8
30	1	1	7	8
35	5	5	10	7
40	2	2	6	6
45	1	1	10	10
50	3	4	9	9

**Table 4:** Best multiples of artificial frames yielded by EMD and EA-EMD at each number of training trials for dataset 2.

Number of training trials	EMD		EA-EMD	
	LDA	LR	LDA	LR
5	1	1	10	8
10	1	3	10	9
15	3	3	7	7
20	1	3	10	7
25	1	1	8	10
30	1	3	9	7
35	1	1	7	6
40	1	1	8	8
45	1	2	10	10
50	1	1	10	8

## Discussions and conclusion

The performance of BCIs improves with the amount of training data available. For the small EEG dataset, it is difficult to use typical paradigm to achieve good classification results. Thereby, a 20–30 minutes calibration phase at the beginning of each use is usually needed to acquire sufficient labeled data for training the subject-specific BCI model. This calibration phase is inconvenient and time consuming, limiting the applicability of a BCI system in real life. Thus, developing reliable methods that reduce calibration time while keeping an acceptable accuracy is highly desired in BCI research.

This paper presents a new method to create EEG artificial data by EA-EMD for reducing the calibration time. The performance of the proposed method for augmenting training set was evaluated on two MI-based BCI data sets and compared with that of EMD applied to raw training set and that of Baseline without the expansion of training set. From the results obtained in this study, we can reach the following conclusions: 1) Applying EMD to raw data is not a valid method for creating artificial EEG trials and expanding the size of training set; 2) Applying EMD to aligned data is indeed an efficient approach for creating artificial frames and reducing the calibration time in BCI systems; 3) EA-EMD can generate more artificial trials than EMD at the same number of training trials and the parameter, multiple of artificial frames, is not sensitive to classification accuracy. These conclusions provide a guideline for creating artificial EEG data with EMD.

From Fig 4(A) and 4(B), it is shown that compared to EMD, the major reason that EA-EMD works is that the artificial frames generated by the appraoch can expand the feature space in a relevant way, in which the two types of features are kept well separated although much more artificial trials are added in the training set. This is conducive to subsequent feature classification.

The proposed approach EA- EMD is able to significantly reduce the calibration time of MI-based BCI systems. From Fig 5, it is observed that using classifiers LDA and LR, EA-EMD achieved an acceptable accuracy of 73% and 74% respectively with only 10 training trials per class, approximately equal to that yielded by Baseline using 30 and 40 training trials per class respectively, amounting to reducing calibration time by 2/3 and 3/4 respectively; From Fig 6, it is observed that using LDA and LR, EA-EMD achieved a good accuracy of 84% and 79.8% respectively with only 10 training trials per class, approximately equal to that yielded by Baseline using 30 and 30 training trials per class respectively, amounting to reducing calibration time by 2/3 and 2/3 respectively.

Figs 7 and 8 demonstrate that the proposed EA-EMD yielded a remarkable increase in the classification accuracy for most subjects that initially have a poor or medium accuracy. However, the observed improvement for a few subjects with initially low BCI performance, for example, subjects 2 and 6 in data set 1, was not pronounced. The reason might be that the genernation of artificial frames for these subjects was not effective since their feature spaces for different classes were not separable. These findings suggest that to increase the accuracy of these subjects with poor subject-specific BCI performance, training set should be augmented with a different approach, e.g. transfer learning.

**Fig 10:**
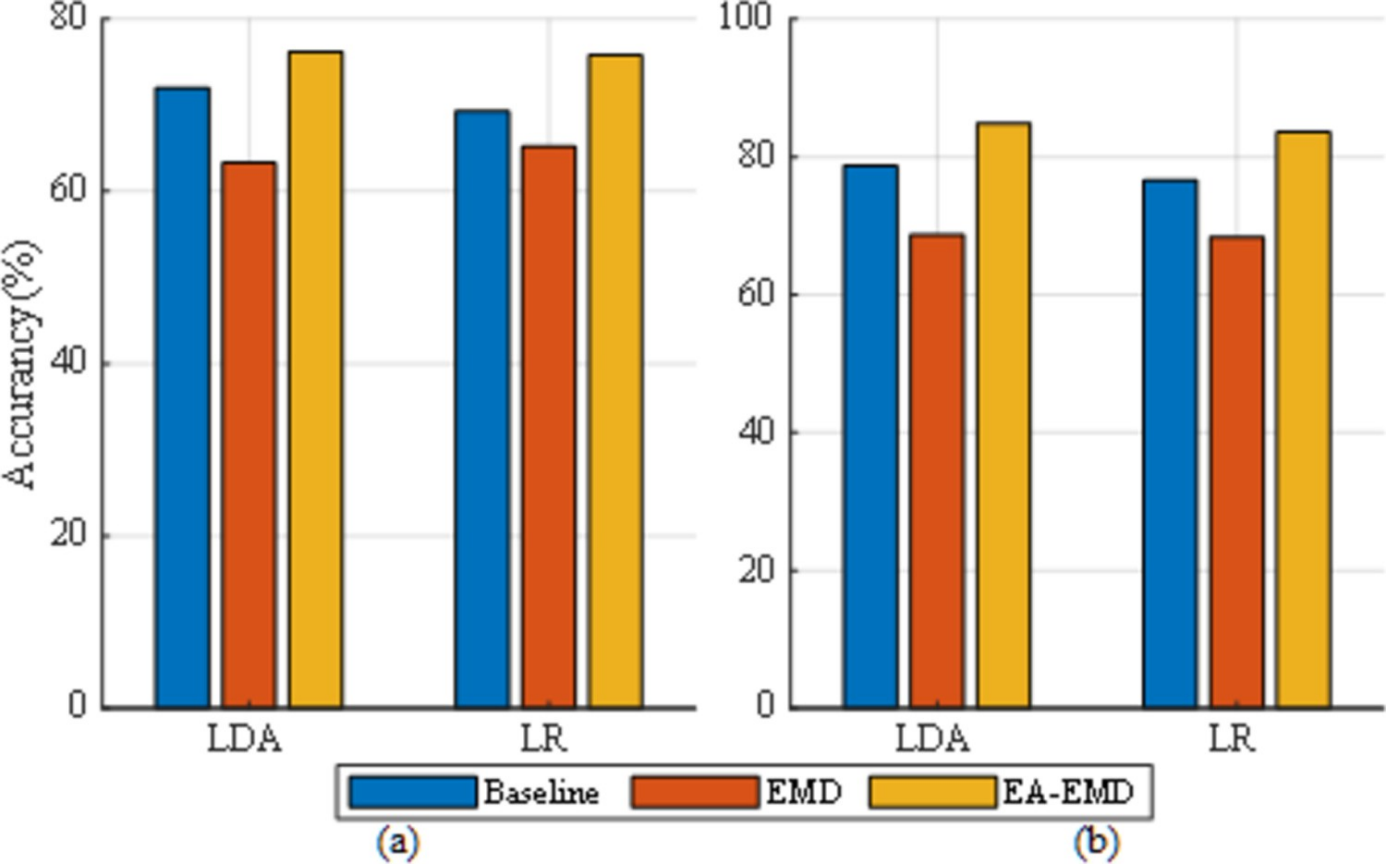
The classification accuracies achieved by training the two classifiers (LDA and LR) with 20 artificial trials (yielded by EMD and EA-EMD) and with 20 real EEG trials (Baseline) for (a) data set 1 and (b) data set 2.

The classification accuracies of training the classifiers with 20 artificial trials per class and with 20 real EEG trials per class are shown in Fig 10. It is observed from the figure that irrespective of classifiers and data sets, the results yielded by artificial data without data alignment are inferior to those yielded by real data, but the results yielded by artificial data with data alignment are superior to those yielded by original data. These results further validate the conclusion drawn in the study, i.e. EA-EMD outperforms Baseline and EMD due to the introduction of EA-based data alignment.

**Fig 11:**
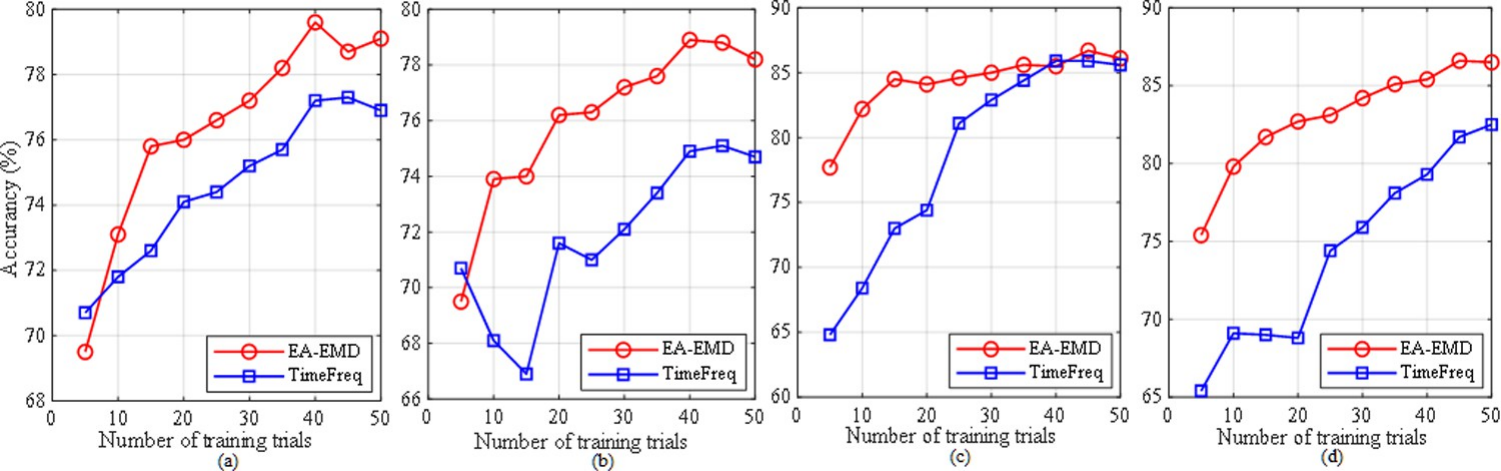
Averaged classification accuracies across all subjects from each data set of two artificial data generation techniques, our p method (EA-EMD) and Lotte’s method (TimeFreq): (a) data set 1 and classifier LDA, (b) data set 1 and classifier LR, (c) data set 2 and classifier LDA, and (d) data set 2 and classifier LR.

Lotte proposed three artificial data generation techniques [22, 23], i.e. generating artificial data by signal segmentation and recombination in time domain and in time-frequency domain, and based on analogy. Among them, the second one (we name it TimeFreq hereinafter) is the best method. We compared the performance of the method TimeFreq with that of our proposed method EA-EMD. The results yielded by the two data sets and the two classifiers are shown in Fig 11. It is observed from the figure that for data set 1, EA-EMD outperforms TimeFreq at all numbers of training trials except for 5 training trials using either classifier; for data set 2, EA-EMD is superior to TimeFreq at all numbers of training trials except for 40 trials using LDA, and outperforms TimeFreq at all numbers of training trials using LR.

In summary, our results suggested that the proposed method for the generation of artificial frames could improve classification accuracy subtantially as compared to the standard BCI design (the Baseline method) paticularly when a small subject-specific training data was available. Importantly, the method not only reduced the required calibration time but also for many subjects they enhanced the classification accuracy, facilitating the real-world application of MI-based BCI systems. This study analyses the proposed algorithm offline. Future work will focus on its implimentation online and other methods for reducing calibration time of BCIs.
